# Chemotherapeutic Drugs: DNA Damage and Repair in Glioblastoma

**DOI:** 10.3390/cancers9060057

**Published:** 2017-05-26

**Authors:** Laura Annovazzi, Marta Mellai, Davide Schiffer

**Affiliations:** Research Center, Policlinico di Monza Foundation, Via Pietro Micca 29, 13100 Vercelli, Italy; marta.mellai@cnbo.it (M.M.); davide.schiffer@unito.it (D.S.)

**Keywords:** glioblastoma, glioma stem cells, DNA damage/repair, chemoresistance

## Abstract

Despite improvements in therapeutic strategies, glioblastoma (GB) remains one of the most lethal cancers. The presence of the blood–brain barrier, the infiltrative nature of the tumor and several resistance mechanisms account for the failure of current treatments. Distinct DNA repair pathways can neutralize the cytotoxicity of chemo- and radio-therapeutic agents, driving resistance and tumor relapse. It seems that a subpopulation of stem-like cells, indicated as glioma stem cells (GSCs), is responsible for tumor initiation, maintenance and recurrence and they appear to be more resistant owing to their enhanced DNA repair capacity. Recently, attention has been focused on the pivotal role of the DNA damage response (DDR) in tumorigenesis and in the modulation of therapeutic treatment effects. In this review, we try to summarize the knowledge concerning the main molecular mechanisms involved in the removal of genotoxic lesions caused by alkylating agents, emphasizing the role of GSCs. Beside their increased DNA repair capacity in comparison with non-stem tumor cells, GSCs show a constitutive checkpoint expression that enables them to survive to treatments in a quiescent, non-proliferative state. The targeted inhibition of checkpoint/repair factors of DDR can contribute to eradicate the GSC population and can have a great potential therapeutic impact aiming at sensitizing malignant gliomas to treatments, improving the overall survival of patients.

## 1. Introduction

Glioblastoma (GB), WHO grade IV glioma [1], is the most common and most malignant primary brain tumor. Even after optimized multimodal treatment with maximal surgical resection, radiotherapy and chemotherapy used in a combinatorial approach [2], the tumor recurs and the prognosis remains very poor with a median survival of approximately 15 months [3,4].

Currently, the drugs of choice for the first line therapy of gliomas and with a proven effectiveness include the methylating agent temozolomide (TMZ) and the chloroethyl-derivatives of nitrosourea: carmustine (BCNU), nimustine (ACNU), lomustine (CCNU) and fotemustine. These drugs cross the blood–brain barrier (BBB) and effectively improve clinical outcomes when used alone or in combination with radiotherapy [3], but, even though they can achieve therapeutic concentrations in the brain, chemoresistance of glioma remains one of the major problems [5].

The incurability of GB is attributable to its profound therapy resistance, due to its genomic and cellular heterogeneity, to its highly infiltrative nature and to several distinct mechanisms that enable the cancer cells to escape radio- and chemo-treatments [6,7]. GBs respond to DNA injuries induced by ionizing radiation (IR) and genotoxic drugs by activating the DNA repair machinery [8,9,10]. Furthermore, tumors are able to eliminate chemotherapeutic compounds from cells through the increased expression and activity of efflux ATP-binding cassette (ABC) transporters, specifically P-glycoprotein (P-gp)/MDR1, MRP1 and BCRP1/ABCG2 [11,12].

## 2. DNA Damage Response (DDR)

The term DNA damage response (DDR) indicates the sophisticated cellular network that senses, signals and repairs DNA insults [13,14]. DNA damage repair in tumors has two distinct opposing aspects: on one side it protects the integrity of genetic material of normal cells, on the other side it contributes to the resistance of tumor-driving cells to genotoxic therapies. At the beginning of gliomagenesis DDR machinery is constitutively activated by oncogene-evoked replication and oxidative stress [15] and acts as a protective mechanism preventing the expansion of malignant clones; however, during transformation, cancer cells can accumulate and tolerate genome damages and rearrangements because of DDR aberrations. The efficiency of DDR is ensured by the redundancy of the pathways [5,16,17].

Since DNA repair systems attenuate the efficacy of genotoxic treatments, understanding and characterizing the repair mechanisms is paramount to develop new therapeutic strategies.

## 3. Glioma Stem Cells (GSCs) and Chemoresistance

**Figure 1:**
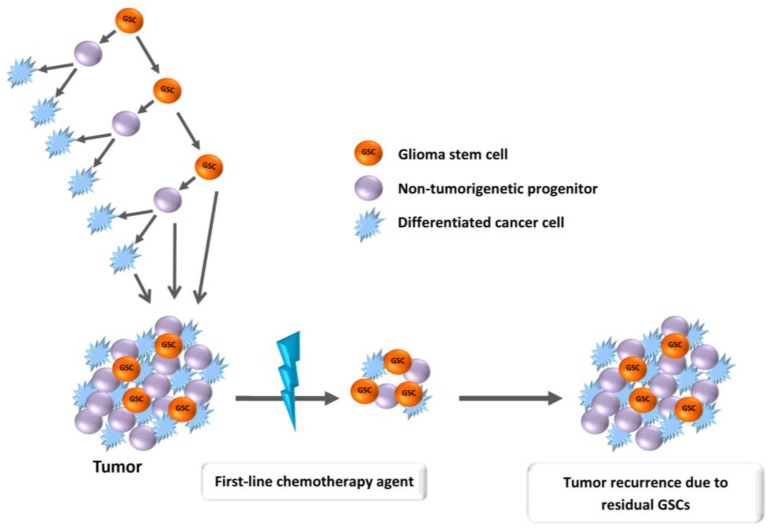
Glioma stem cell (GSC) model of chemoresistance. GSCs are relatively resistant to standard chemotherapy and are responsible for tumor relapse.

Within the heterogeneous tumor mass of GB, a subpopulation of cells exists designated glioma stem cells (GSCs), showing high similarities with neural stem cells [18]. They are characterized by long-term proliferation, self-renewal, multi-lineage differentiation potential in vitro and tumorigenic capability in vivo [19,20,21]. Several studies indicated that GSCs represent the cells able to persist after standard treatment and responsible for tumor cell repopulation during recurrence [22,23,24,25] (Figure 1). According to this hypothesis, the failure of current chemotherapies to eliminate the bona fide stem cells is the reason for chemoresistance. It seems that GSCs possess a superior DNA repair profile compared to non-stem tumor cells [26,27,28]. Therefore, efforts to improve chemotherapy response should be directed specifically to hit this subset of tumor cells [29].

The susceptibility of tumor cells can be modified by the microenvironment and by auto- and paracrine signaling generated by tumor cells or by invading non-tumor cells like microglia [30,31]. Thus, differentiated cells can acquire stemness properties under hypoxic condition [32,33,34] or after sublethal concentrations of chemotherapy [35]. The existence of a kind of cell plasticity has been hypothesized, with the possibility of conversion of non-GSCs into GSCs [36,37] and this makes it even more difficult to target the cell population responsible for malignant relapse.

However, it is still a matter of question whether GSCs are actually more resistant to chemotherapy in comparison to non-stem cells [27,28,38]. The lack of reliable stem cell markers for in vitro and in vivo research makes it arduous to answer this question. CD133/Prominin is a cell surface marker expressed by neural stem cells and initially used to identify and characterize the population of cancer stem cells in GB [20]. CD133+ cells isolated from resected tissues are able to generate neurospheres (NS) in culture, to express markers of the three neural lineages in differentiation conditions and to induce brain tumor in xenograft models [21]. However, subsequently, CD133− cells with stemness properties and tumorigenic potential have been isolated [39]. Thus, the specificity of CD133 as a marker to unequivocally identify GSCs remains questionable.

It has been reported that GSCs maintained under serum-free culture conditions displayed an increased resistance to chemotherapy as compared to tumor cells cultured under conditions favoring differentiation [40,41]. It has been shown that the DNA damage checkpoint response was constitutively activated in CD133+ cells isolated from GBs, with checkpoint kinases (Chk1 and Chk2) phosphorylated even in absence of treatment. Moreover, in response to radio-treatment, CD133+ cells repaired IR-induced DNA damage more efficiently than CD133− cells and the radioresistance could be reversed with specific inhibitors of Chk1 and Chk2 [26]. Increased basal activation of checkpoints in untreated GSCs was confirmed by Ropolo et al. [42], suggesting that this condition may contribute to treatment resistance by determining a cell cycle delay that confers GSCs more time for removal of radio- and chemotherapeutic-induced DNA lesions before replication [43]. The possibility exists that only a quota of CD133+ tumor cells actually represent GSCs and it has been also supposed that the population doubling time of GSCs was significantly longer compared to non-stem cells; thus, it has been proposed that slow proliferation rate and an elongated cell cycle may be a major mechanism for cells to resist genotoxic therapies [42,44,45,46]. The ability to assume a quiescent cell status may explain the greater repair capacity and resistance to treatments of cancer stem cells in comparison to proliferative cells [47,48]. The effect of DNA damaging drugs on GSCs would be, therefore, cytostatic rather than cytotoxic. In the literature, there is no agreement on the efficacy of chemotherapeutic drugs on GSCs. From in vivo and in vitro experimental data, it is controversial whether tumor stem cells are actually more resistant to chemotherapy in comparison with more differentiated tumor cells. As regards in vitro experiments, the reason of these contrasting results is due to the employment of different culture conditions (Neurosphere Assay with growth factors vs. medium with serum) and different drug dosing schemes. It has been reported that GSCs cultured as NS in serum-free medium proved more resistant than GSCs grown under culture conditions favoring differentiation [40,41]. It should be mentioned that NS cultures more closely reflect the genotype and the phenotype of the tumor of origin and would represent a more valid in vitro model of GB [49]. It has been proved that chronic treatment with therapeutic doses of TMZ increased the quota of CD133+ tumor stem cells, both in vitro and on xenografted specimens, and induced stemness in differentiated tumor cells [35,50]. In TMZ-treated cells a phenotypic shift of differentiated non-GSCs toward a stem-like state was observed. This converted population of GSCs expressed stemness markers, such as CD133, SOX2, Oct4 and Nestin, and when implanted orthotopically in nude mice, resulted more tumorigenetic, originating a more efficient grafting and invasive phenotype [35]. Bleau et al. demonstrated that, in a murine model of GB, long-term treatment with subtoxic TMZ doses enriched the side population of cancer stem cells, increasing the tumor aggressiveness [51]. Similar findings are reported by Pallini et al. that found an increase of stem cell marker CD133 up to 20× in recurrent glioblastomas after radio-chemotherapy [52]. A recent in vitro study [53] showed that two cycles of TMZ treatment resulted in the increase of TMZ resistance in GB cell lines not expressing the repair enzyme O^6^-methylguanine-DNA methyltransferase (MGMT); at the same time the drug caused a significant increase in the CD133+ population, confirming that TMZ can promote the generation of GSCs.

Conversely, some studies showed an increased rather than decreased sensitivity to TMZ of GSCs in comparison to differentiated tumor cells and agreed on the efficacy of TMZ pulse treatment to inhibit the clonogenic growth of GSCs in vitro, without, however, killing the cells [38,54,55]. Nevertheless, almost all GB tumors relapse after multimodal therapy suggesting that residual GSCs survived in vivo; it can be hypothesized that, owing to the presence of the BBB, the TMZ concentrations achieved in patients are only sufficient to completely eliminate GSCs in vitro from MGMT-negative but not from MGMT-positive tumors.

An in vitro study reported that BCNU increased the proportion of tumor stem cells, in terms of CD133 expression and NS growth ability [56].

The lack of a unique and unambiguous stem cell marker does not allow to reach a final answer on the effectiveness of alkylating drugs on GSCs. In GSCs, a complex interaction exists between the activation of phosphatidylinositol-3-kinase (PI3K)/Akt signaling, frequently upregulated in gliomas, the loss of tumor suppressor phosphatase and tensin homolog (PTEN) and therapy resistance. Akt inhibitors or induction of PTEN expression can reverse the resistance and sensitize cells to chemo- and radiotherapy by impairing DNA repair [57,58,59].

## 4. Double Strand Break Repair

**Figure 2:**
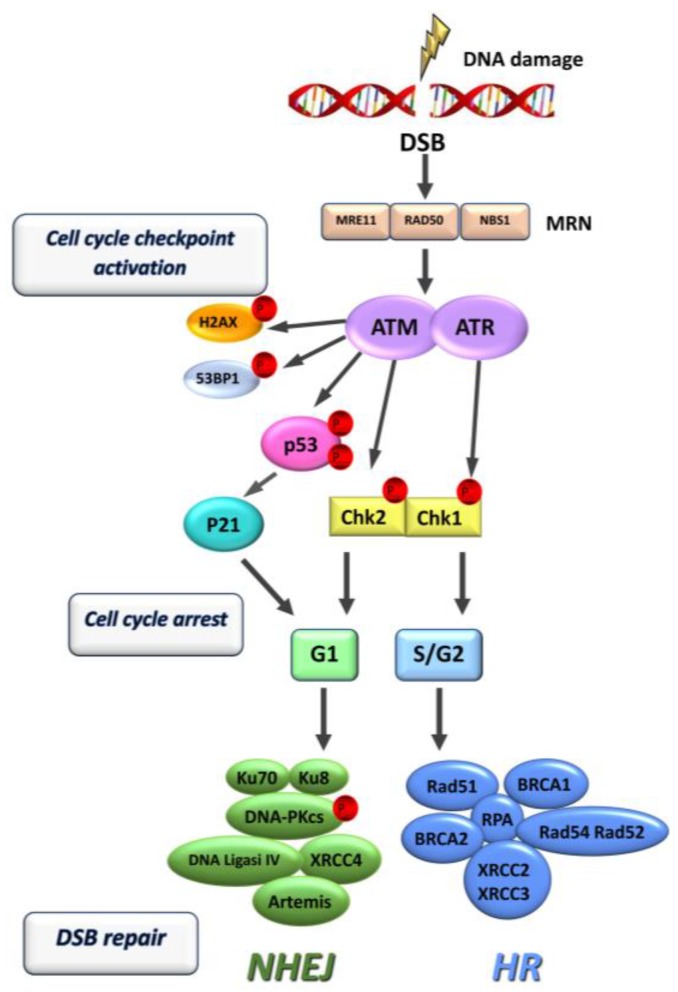
Scheme of cell checkpoint/repair signaling after DNA double strand break (DSB) formation (modified from Bolderson et al. [63]).

The most severe DNA lesions for a mammalian cell are double strand breaks (DSBs), that may be induced by IR, genotoxic drugs and, indirectly, by endogenous reactive oxygen species. DSBs are produced as ultimate lesions following damage induced by alkylating agents. Specifically, the primary DNA alkylation damage, causing base mispairing, is converted by mismatch repair (MMR) into critical secondary pre-apoptotic lesions that induce replication fork collapse and, finally, DSBs triggering the DDR [60,61,62] DSBs can be repaired by two mechanisms: homologous recombination (HR) and non-homologous end joining (NHEJ). In response to a DNA injury, cells activate a temporary arrest of cell cycle allowing for processing of damage before arrival of the replication fork. This response is started by sensing DSB damage by the Mre11/Rad50/Nbs1 (MRN) complex with subsequent activation of the ataxia telangiectasia mutated (ATM) and Rad3-related (ATR) kinases that trigger the phosphorylation of downstream targets involved in cell cycle arrest, DNA repair and apoptosis. Key targets of ATM include H2AX histone, a specific indicator of DSB occurrence, 53 binding protein 1 (53BP1) and the checkpoint kinases 1 (Chk1) and 2 (Chk2); these last two determine the arrest of cell cycle in G1/S, intra-S or G2/M phases [45]. ATM determines the phosphorylation of the tumor suppressor protein p53, which in turn activates p21, leading to the cyclin-dependent inhibition of cycle progression [63]. If repair fails, due to severity of damage, apoptosis is triggered [64,65]. HR is a high-fidelity repair system and takes place only in actively cycling cells; it requires a complementary undamaged sister chromatid of DNA as a template, that is available only after DNA replication in S phase. HR proteins include members of the MRN complex as well as Rad51, XRCC2 and XRCC3 [66]. The products of the breast cancer susceptibility genes, BRCA1 and BRCA2, are also involved in the HR pathway. NHEJ occurs predominantly during G0 and G1 phases of the cycle and repairs the broken DNA ends without a template, not preserving the original genetic information and causing possible errors in the DNA sequence. Proteins involved in the NHEJ pathway include the DNA dependent protein kinase (DNA-PK), which consists of a regulatory subunit (Ku70/80) and of a catalytic subunit (DNA-PKcs); the other NHEJ effectors are DNA ligase IV (Lig4), XRCC4 and Artemis (Figure 2).

It has been observed that the expression of DDR proteins is higher in glioma than in the adjacent normal tissue and ATM expression levels have been proposed as an independent prognostic factor related to longer survival [67].

A direct interaction between epidermal growth factor receptor (EGFR), a receptor tyrosine kinase amplified in about 40% of GB patients, and DNA repair has been observed. EGFR signaling has been shown to activate, through Akt and MEK/ERK, both HR and NHEJ systems. Upregulation of wild-type (wt) EGFR or expression of the EGFR variant III (EGFRvIII) have been associated in U87 glioma cells with promotion of γ-H2AX foci resolution, a surrogate for DSB repair, and with the nuclear accumulation of ATM, DNA-PKcs and RAD51, essential for the activity of NHEJ and HR. Moreover, blocking EGFR signaling with small molecule inhibitors or the expression of dominant-negative EGFR (EGFR-CD533) have been proved to significantly attenuate the DDR [68]. Amplification of EGFR and expression of EGFRvIII mutation have been reported to correlate with an enhanced DSB repair response and resistance to treatments in GB patients [69,70].

## 5. Resistance Mechanisms to Alkylating Agents

DDR pathways are involved in the repair of damage induced by both TMZ and chloroethyl-nitrosoureas (CNUs) [60,61,71,72].

**Figure 3:**
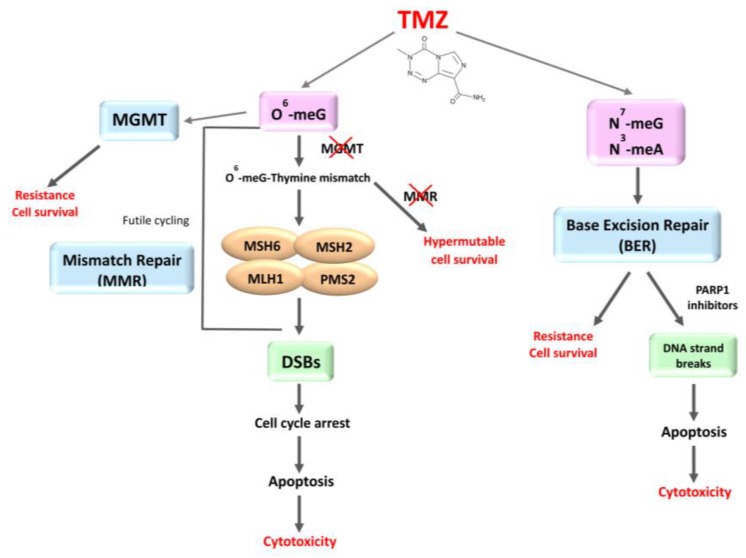
Mechanisms of cytotoxicity of temozolomide (TMZ) and repair systems of resistant cells.

The alkylating agent TMZ leads to the formation of a wide spectrum of methyl adducts, especially N-methylpurines, which are easily repaired by base excision repair (BER) system with the involvement of poly (ADP-ribose) polymerase-1 (PARP-1). The cytotoxicity of TMZ is mainly due to the O^6^-methylguanine (O^6^-meG) adducts, that are normally removed by MGMT in a suicide reaction that irreversibly inactivates the enzyme. If MGMT is deficient, the unrepaired O^6^-meG mispairs with thymine triggering a futile MMR cycle with consequent DSB formation, activation of ATM/Chk2 signaling and induction of p53-associated G_2_/M cycle arrest [73]. TMZ is reported to provoke autophagy, mitotic catastrophe, senescence and apoptosis [74,75] (Figure 3).

The major mechanism of TMZ resistance in GB patients is represented by MGMT expression. Hypermethylation of the promoter region of the MGMT gene, found in 30–60% of GB patients [76], determines epigenetic transcriptional silencing of the protein and correlates with a better clinical response to alkylating agents associated with prolonged survival [77,78]. Thus, from the levels of MGMT it is possible to predict the response to TMZ in GB patients [79,80]. It was reported that in MGMT-deficient GBs, TMZ increases the DSB lesions caused by radiotherapy [44,81]. The mechanisms of TMZ-induced growth inhibition of glioma cells are controversial. According to Roos et al. [82], the cytotoxic effect of TMZ depends on apoptosis, but some reports suggested that induction of senescence plays a main role [75,83,84] and is mediated by the permanent presence of DSBs. Beside MGMT expression, major determinants in the response to TMZ are MMR system and p53 status [73,79,82,85,86]. Defects in MMR [87,88] or BER [5,89] and p53 mutation [79,90] are involved in TMZ resistance of glioma cells, even in absence of MGMT (Figure 3). The integrity of MMR system, and specifically of MSH2/MSH6 dimers, is fundamental to induction of cytotoxicity in TMZ treatment, and several studies in experimental models indicated that, if MMR is not functional or defective, tumor cells become resistant to the killing effects of alkylating agents. TMZ induces cell cycle arrest in G2/M phase and cell death in MMR-proficient but not in MMR-deficient cells [74]. Thus, in gliomas with MGMT methylation, after TMZ treatment, cells with MMR deficiency, instead of entering apoptosis, continue to replicate with genetic alterations otherwise inconsistent with progression of cell cycle, becoming therapy-resistant and potentially developing additional mutations in resistant clones [88,91,92] (Figure 3). Essentially all GBs recur after alkylating agent chemotherapy becoming refractory to further treatment.

A recent work demonstrated in 20% of recurrent GB patients the occurrence of a hypermutated genotype, which can be acquired stochastically or as result of treatment with mutagen agents, such as alkylating agents [93]. It has been shown that chronic exposure of a GB line to TMZ produced resistant clones harboring MSH6 mutations and that MSH6 reconstitution restored sensitivity to the drug in MSH6-null cells [94]. Gliomas treated with TMZ acquire somatic MSH6 mutations, conferring TMZ resistance and resulting in a hypermutational process, which facilitates rapid evolution of clones with growth advantage and characterized by a high mutational and neoantigen load [90]. The hypermutant genotype was only found out at recurrence and was always associated with mutations or decreased expression of MMR genes, most commonly in MSH6, that were absent in pretreatment tumors [92,93]. The derived intra-tumor heterogeneity, which is the fraction of clonal and subclonal neoantigens, therefore, plays an important role in the response to treatments [95,96]. In the last few years some studies have put into evidence the clinical relevance of the hypermutation genotype. In fact, a high mutational burden entails the fact that some neoantigens become visible to the immune system and potential targets of the T cell response. Recently it has been demonstrated that tumors harboring hypermutations may stronger respond to immunotherapy with checkpoint inhibitors, such as the anti-programmed cell death-1 (PD-1) [97,98]. It has been reported that hypoxia can induce MGMT expression in GSCs, promoting chemoresistance [30]. On the other side, overexpression of wt p53 can suppress MGMT activity and make glioma cells more sensitive to TMZ [99]. In this study, p53 accumulation resulted in the loss of MGMT mRNA, protein and enzyme activity and induction of wt p53 led to a three- and twofold increase in sensitivity to BCNU and TMZ, respectively. Moreover, a panel of four human tumor cell lines, including gliomas, with wt p53 status, displayed markedly lower levels of MGMT gene transcripts in comparison with p53 mutated cells. These results demonstrated that p53 is a negative regulator of MGMT gene expression in human tumors.

It has been shown that both ATM and ATR contribute to the resistance of GB cells to TMZ in vitro [62,100]. The use of the specific ATM inhibitor KU-60019 has been proven to reduce DDR, glioma cell migration and invasion by downregulating basal activation of Akt [101].

Several studies have linked TMZ sensitivity to efficacy of HR system [102]. Silencing of BRCA2 was found to increase the cytotoxicity of TMZ in GB cells deficient in MGMT, indicating a role of HR in resistance to TMZ [61]. Moreover, Quiros et al. demonstrated that silencing RAD51 in combination with a PARP-1 inhibitor improves the cytotoxicity of TMZ in MGMT-deficient LN-229 GB cells, whereas hampering NHEJ through pharmacological inhibition of DNA-PK did not change significantly the cytotoxicity of the drug [103]. The importance of HR in repairing TMZ lesions in the absence of MGMT was confirmed by Short et al. [104]. Recently, Nagel et al. [105] showed that HR contributes to acquired TMZ resistance in mouse orthotopic xenograft models of GB, leading to reduced survival of TMZ treated animals. However, it was observed that siRNA-mediated depletion of the NHEJ factor DNA ligase IV sensitizes cells of A172 glioblastoma line to TMZ [72]. In 53% of surgical samples of GB, RAD51 levels have been found higher within the tumor compared to the normal surrounding tissue [106] and correlated with longer survival and better prognosis. This finding suggests that RAD51, would be an index of genetic instability, rather than of repair activity, and would mark cells more susceptible to genotoxic injuries.

CNUs are powerful agents used in cancer therapy that alkylate the DNA molecule on different sites, mainly at the N^7^-position of guanine and the N^3^-position of adenine. The main killing lesion is the O^6^-chloroethylguanine, that can be repaired by MGMT. Otherwise, this adduct is unstable and undergoes spontaneous intra-molecular rearrangements to form an intermediary N^1^-deoxyguanine-N^3^-deoxycytosine producing highly toxic inter-strand cross-links (ICLs). The unrepaired ICLs block replication fork finally resulting in the formation of DSBs subject to HR or NHEJ or in cell death [72,107,108,109]. Contrary to what observed for TMZ, chloroethylating drugs are more toxic in p53-mutated than in wt p53 glioma cells. In fact, a functional p53 seems to stimulate the repair of DSBs generated by CNU lesions, protecting glioma cells against the killing effects of these drugs [107].

## 6. In Vitro Cytotoxicity Study on Neurospheres (NS) and Adherent Cell (AC) Cultures

**Figure 4:**
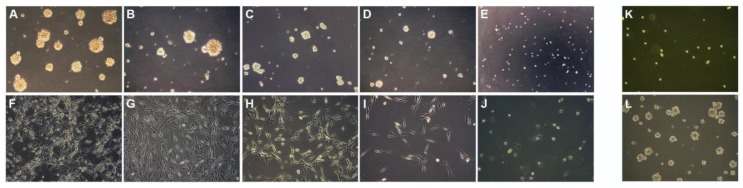
Cytotoxic effect of TMZ, DOX and PTX on glioma cells. Inhibitory effect of TMZ increasing doses (5, 50, 100, 200 μM) at 72 h: on NO3 NS (**B**–**E**); and on NO3 AC (**G**–**J**) growth; untreated NS and AC in (**A**,**F**), respectively; few surviving cells of NO3 NS line after 120 h treatment with 200 μM TMZ (**K**); and proliferation restarting after 30 days from treatment suspension (**L**). All 100× magnification. Reproduced with permission from Annovazzi et al., International Journal of Oncology; published by Spandidos Publications Ltd., 2015 [110].

In a recent work [110], we analyzed the effect of TMZ and other two commonly used anticancer drugs, doxorubicin (DOX) and paclitaxel (PTX), on 16 primary cell lines established in our laboratory from GB surgical samples. DOX and PTX use in brain tumor therapy is currently limited because of their poor capacity of cross the BBB, even though their effectiveness on glioma cells has been proven in vitro and in animal models [111,112,113]. These drugs are reported to induce DNA strand breaks [114,115]. We demonstrated that the three drugs can reduce significantly cell viability, inhibiting dose- and time-dependently cell proliferation and clonogenic growth, both of NS cultures, maintained on serum-free medium with growth factors, and of AC cultures, maintained on serum-medium (Figure 4). The effect of TMZ and DOX was, however, more evident on AC than on NS. With the range of concentration employed, DOX and PTX showed greater cytotoxicity in comparison with TMZ. As regards TMZ, the susceptibility of cells depended on the status of MGMT and p53: among the cell lines presenting hypermethylated MGMT, those with a wt p53 gene appeared more sensitive than the ones with mutated p53.

**Figure 5:**
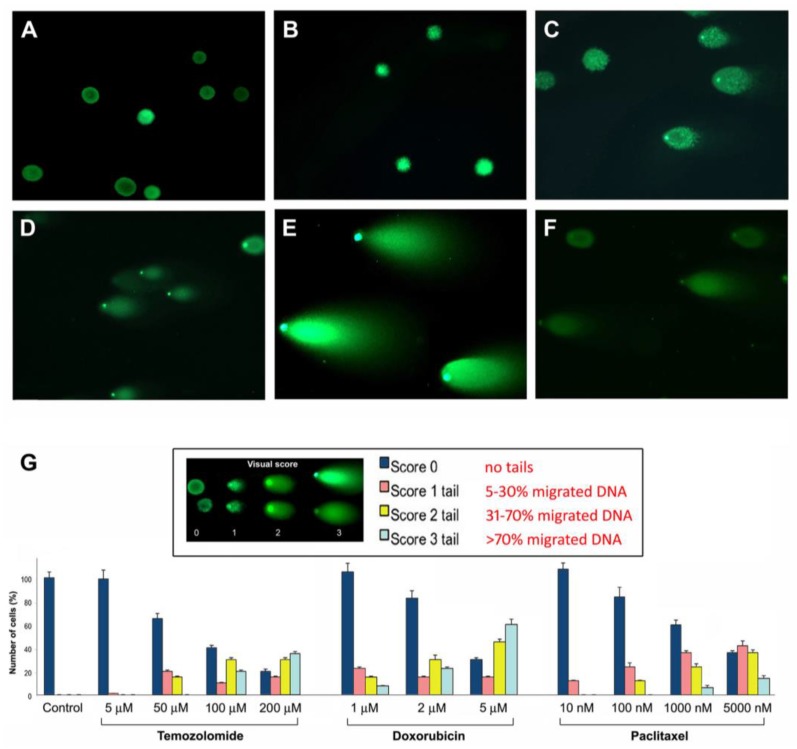
Comet assay on NO3 NS line. Comet assay on: untreated NO3 NS (**A**); NS after 50 μM TMZ for 24 h (**B**); NS after 50 μM TMZ for 72 h (**C**); NS after 200 μM TMZ for 72 h (**D**); NS after 2 μM DOX for 72 h (**E**); and NS after 100 nM PTX for 72 h (**F**). Quantification of DNA damage in untreated cells (control) of NO3 NS and after 72 h treatment with TMZ, DOX and PTX at various doses (**G**), by using an arbitrary visual score (upper panel); data are mean values ± SE of three independent experiments, each performed in triplicate. Reproduced with permission from Annovazzi et al., International Journal of Oncology; published by Spandidos Publications Ltd., 2015 [110].

**Figure 6:**
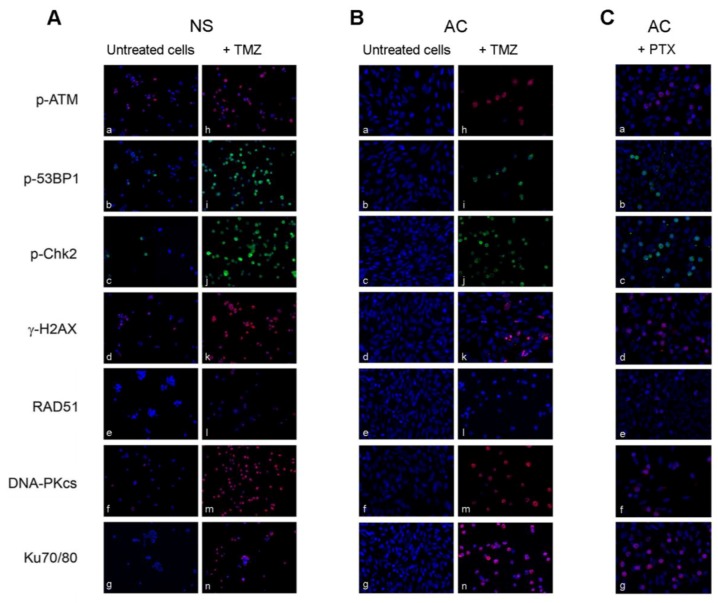
Study of checkpoint/repair response in NO3 NS and AC. (**A**) Expression by immunofluorescence of checkpoint/repair proteins: in untreated NO3 NS (**a**–**g**); and in cells treated with 100 μM TMZ for 48 h (**h**–**n**). (**B**) The same as above in: untreated (**a**–**g**) and treated NO3 AC (**h**–**n**). (**C**) The same as above in NO3 AC treated with 100 nM PTX for 72 h (**a**–**g**): positivity of the antigens is evident at metaphasis level. Nuclei counterstained with DAPI. All 200× magnification. Reproduced with permission from Annovazzi et al., International Journal of Oncology; published by Spandidos Publications Ltd., 2015 [110].

By Comet assay, it was observed that all the three antineoplastic agents can provoke a genotoxic damage, that increased proportionally with the concentration of the drugs and with the time of exposure (Figure 5). The first cell response to DNA lesions, observed already after 48 h treatment, in NS and, at a minor extent, in AC lines, was the activation of the sensor molecules (ATM, γ-H2AX, 53BP1, Chk2) followed by repair effectors (Ku70/80, DNA-PKcs, RAD51) (Figure 6). From the data of protein expression by immunofluorescence and Western blot analysis, it appeared that DNA repair was accomplished mainly through NHEJ pathway and, much less, through HR pathway. Interestingly, we noted in some untreated NS lines a moderate basal expression of some DDR proteins (p-ATM, Ku70/80, DNA-PKcs). Expression of the molecules of repair cascade resulted, in average, higher in NS than in AC lines, indicating that the former, containing GSCs and progenitors, were more resistant to the treatments than the latter, more differentiated cells. Interestingly, it has been observed that conditions of reduced cell adhesion, which allow tumor cells to migrate and spread, significantly affect sensitivity to DNA damage and decrease cell death; suspended cells show more resistance to genotoxic treatments. In particular, in certain cell types loss of integrin-mediated adhesion has been reported to cause a decline of p53 decreasing the apoptotic response to genotoxic chemotherapeutic agents. In p53-negative tumor cells, response to DNA damage could be restored activating the alternative pathway of c-Abl tyrosine kinase and p73 that regulates integrin-mediated adhesion [116]. It was observed, moreover, that a relationship exists between the cytoskeleton dynamics regulating cell motility and p53 response upon DNA injury. In DNA damage conditions, nuclear accumulation of the transcription co-factor JMY (junction-mediating and regulatory protein) has been shown in some cell types to augment p53 activity and, at the same time, to limit cell motility and to induce adhesion through regulation of actin and cadherins [117]. These findings are relevant to tumor cells both in vitro, where different culture conditions can be used, and in vivo, where adhesiveness can be compromised owing to the degradation of extracellular matrix or to integrin alterations.

In GB tissue samples, we observed a constitutive expression of the checkpoint/repair proteins, not found in low-grade gliomas [110].

The most important finding was that even high TMZ doses did not lead to the total destruction of stem cells/progenitors; in fact, only a small percentage of cells, especially in NS cultures, underwent apoptosis, whereas, according to the data of Beier et al. [50], a quota of cells survived in a quiescent state and could re-acquire proliferation capacity after drug removal. PTX and mainly DOX effects on cell viability were more irreversible, suggesting that, once the way to overcome the BBB is found, these drugs can be effective cytotoxic agents against glioma cells. For this purpose, we are studying new nanoparticle systems able to efficiently vehiculate hydrophilic compounds across the BBB to the site of tumor [118,119].

## 7. Conclusions

Recent experimental data, elucidating the in vitro behavior of GSCs in response to genotoxic drugs, showed that, on the one side, these cells possess an increased repair capacity in comparison with non-stem tumor cells, while, on the other side, they can assume a non-proliferative state of quiescence, that enables them to contrast the cytotoxicity of antineoplastic agents. Investigating and understanding the mechanisms of chemoresistance in glioma cells is of fundamental importance to develop new effective treatment strategies, since modulation of DNA repair capacity can be a means to increase cellular sensitivity to genotoxic agents. Therefore, controlled targeted inhibition of the DDR factors such as ATM, checkpoint kinases and DNA-PK, combined with chemotherapeutic drugs would represent a useful strategy to prevent temporary cell cycle arrest and DNA damage repair, to promote cancer cell death and to improve patient outcome.
